# The 'Brain Drain' of physicians: historical antecedents to an ethical debate, c. 1960–79

**DOI:** 10.1186/1747-5341-3-24

**Published:** 2008-11-10

**Authors:** David Wright, Nathan Flis, Mona Gupta

**Affiliations:** 1 History of Medicine Unit, HSC 3N10, McMaster University, 1200 Main Street West, Hamilton Ontario L8N 3Z5, Canada; 2 Faculty of Modern History, c/o Linacre College, St. Cross Road, Oxford, OX1 3JA, UK; 3 Women's College Research Institute, 790 Bay St, 7th Floor, Toronto, Ontario, M5G 1N8, Canada

## Abstract

Many western industrialized countries are currently suffering from a crisis in health human resources, one that involves a debate over the recruitment and licensing of foreign-trained doctors and nurses. The intense public policy interest in foreign-trained medical personnel, however, is not new. During the 1960s, western countries revised their immigration policies to focus on highly-trained professionals. During the following decade, hundreds of thousands of health care practitioners migrated from poorer jurisdictions to western industrialized countries to solve what were then deemed to be national doctor and nursing 'shortages' in the developed world. Migration plummeted in the 1980s and 1990s only to re-emerge in the last decade as an important debate in global health care policy and ethics. This paper will examine the historical antecedents to this ethical debate. It will trace the early articulation of the idea of a 'brain drain', one that emerged from the loss of NHS doctors to other western jurisdictions in the 1950s and 1960s. Only over time did the discussion turn to the 'manpower' losses of 'third world countries', but the inability to track physician migration, amongst other variables, muted any concerted ethical debate. By contrast, the last decade's literature has witnessed a dramatically different ethical framework, informed by globalization, the rise of South Africa as a source donor country, and the ongoing catastrophe of the AIDS epidemic. Unlike the literature of the early 1970s, recent scholarship has focussed on a new framework of global ethics.

## Background

The recruitment of health care practitioners from developing to developed countries is now an important topic in global health ethics [1-8]. The intense public policy interest in foreign-trained doctors and nurses, however, is not new. During the mid-1960s, most western countries revised their immigration policies to focus on highly-trained professionals. These immigration changes facilitated the migration of hundreds of thousands of health care personnel from poorer jurisdictions to western countries to solve what were then deemed to be national physician and nursing shortages. Although we are now beginning to understand the broad socio-geographical impact of this massive international migration of health care workers [9-14], little has been written about the historical origins of this important era of post-war medical migration [15,16].

This paper will examine the emergence of the debate over what is now popularly called the "Brain Drain" – the migration of physicians from developing to developed countries and between industrialized nations. It will demonstrate how the early scholarship on the brain drain arose not from a concern over the impact on developing countries, but from a recognition in Britain of the loss of post-war NHS physicians to North America. Occasionally, early research acknowledged that the migration of health human resources from developing to developed countries (which was also occurring apace) raised concerns. However, writings in immigrant receiving countries – such as Canada, the United States, Britain or Australia – did not conceptualize physician immigration as ethically problematic. The responsibility for such a transfer of what was then commonly referred to as 'highly skilled manpower' was understood as the accumulation of thousands of defensible individual decisions made by the doctors themselves. Indeed, much of the literature emphasized the value of advanced medical training being provided by industrialized countries. Moreover, since so much of the medical migration in this period occurred between developed countries the ripple effect on third and fourth countries was seldom fully appreciated or commented upon.

By contrast, the literature over the last decade has witnessed a dramatically different conceptual framework, informed by globalization, the rise in South Africa as a leading 'donor' country, and the ongoing catastrophe of the AIDS epidemic. Unlike the literature a generation ago, new scholarship has focussed on the responsibility (financially or otherwise) of receiving countries to donor countries. Such ideas reflect in part, the rise (and partial acceptance) of international treaties (such as the Kyoto Accord) whereby countries have obligations to the global community for policy decisions they make domestically. This paper explores the historical antecedents to this important ethical debate in global health care.

### The transnational migration of physicians, c.1960–79

By the early 1960s, governments in western industrialized nations recognized with alarm that the domestic production of professionals – university professors, engineers, scientists – was insufficient to provide the same level, let alone a surging demand, for professional services within their respective societies. Nowhere was this more acutely felt than in the domain of health care where rising affluence and technological advances in the treatment of diseases led to a growing need for medical personnel. In English-speaking Commonwealth countries, this demand for health care services was accelerated by the advent and extension of universal state-run health insurance systems which unleashed a seemingly insatiable appetite for state-funded procedures [17-19]. Western, industrialized English-speaking countries were thus to experience in the 1960s and 1970s an acute problem of access to physicians which would be characterized, by the press, as national doctor 'shortages'.

Although precise figures remain difficult to ascertain, western industrialized countries in the 1960s and 1970s licensed an extraordinary number of physicians who were trained outside of their national boundaries. The United States alone accepted over 60,000 foreign-medical graduates (FMGs) between 1963 and 1979 [11]. Canada admitted 12,000 International Medical Graduates (IMGs) between 1961 and 1975 [15]. Between 1966 and 1974, Great Britain licensed 12,640 foreign-trained physicians, a number not including those from the Irish Republic [20]. One World Health Organisation study estimated the net loss of physicians from 'developing countries' to 'developed countries' to be 70,000 in the calendar year 1972 alone [21]. Precise global figures for the period 1960–79 are impossible to obtain, due to the problem of double counting (physicians who migrated to one country, only to depart for another a few years later). But one can realistically assume that doctors who moved from poorer countries to richer countries in this twenty year period numbered in the hundreds of thousands (see below).

Physician migration appears to have peaked in the years 1966–75, and then slowly abated in the late 1970s. Many countries ultimately established new medical schools so as to increase the domestic supply of medical personnel in order to reach the goal of 'self sufficiency' [9,15]. By the end of the decade, and into the 1980s, many health policy experts began to wonder whether their countries had *too many *doctors [9-11]. The international migration of foreign-trained doctors continued throughout the 1980s and 1990s, but at a very much reduced level, and often to support specific programs in rural and/or remote regions. By the 1980s, health policy discussions would shift to how western countries should recognize the credentials of IMGs who were already resident in their own countries [16].

Nevertheless, the impact of the period 1965–79 had been profound. In 1972, 140,000 of the world's physicians were found in countries other than their native ones, and three-quarters of these 140,000 physicians were living in the United States, the United Kindom, Canada, the Federal Republic of Germany, and Australia [21]. By the early 1980s, approximately one third of all licensed physicians in Australia, New Zealand and Canada had been trained outside their adopted countries. Britain reported a quarter of its physician workforce as foreign-trained; the United States one fifth [11].

### The 'brain drain' of physicians from Britain to North America

The term 'brain drain' appears to have become popularized in the context of a substantial body of work about the impact of physician migration on countries in the *developed *rather than developing world. Mainly written by American and British scholars, this literature was the first to address the impact of medical migration on the health systems of the 'first world'. In *Geographical Mobility and the Brain Drain *[22], McKay characterised the term 'brain drain' as a 'peculiarly British invention' that was coined in the mid-1950s (by the Royal Society) to capture the social and professional impact of British medical graduates leaving the country to seek opportunities in North America. McKay's study traced large numbers of Scottish medical graduates who flocked south to England, across the Atlantic to the United States and Canada, and down under to Australia and New Zealand [22].

One of the first major investigations of the migration of English physicians – *British Doctors at Home and Abroad *– was published in 1964 by Abel-Smith and Gales [23]. By interviewing about 3,600 doctors it was surmised that the National Health Service in England was kept afloat during the late 1950s largely thanks to a steady inflow of doctors from Scotland and Wales, a migration that compensated for the great outflow of English medical personnel to North America and elsewhere. The sample also indicated that the emigration from England was increasing (during the late 1950s and early 1960s) at an alarming rate. *British Doctors at Home and Abroad *was less helpful when it came to the question of why doctors were leaving England, and it included no information whatsoever about physicians born outside of the United Kingdom and the Irish Republic who also lent their support to the British National Health System from 1950s onwards. In fact, from about the mid 1960s, England's physician supply was becoming heavily dependent on non-British and non-Irish sources. By 1966, for example, 8785 physicians from the 'developing world' were working in Britain, 70% of whom were from the Indian sub-continent. Yet no official acknowledgement of their contribution appeared in the literature until the Royal Commission on the National Health Service's survey entitled *Doctor Manpower *in 1978 [20].

American scholars were equally concerned about an emerging 'doctor shortage' in the late 1960s. Rashi Fein described, in terms of 'social economics', the doctor shortage that affected the United States in the mid-1960s, and suggested that increasing the output of American medical graduates was but one way of addressing the then present and future demand for physicians. According to Fein, increased reliance on medical auxiliaries would also do a lot for the United States. Fein argued that it was the responsibility of the United States as a leading world power to encourage foreign medical graduates to return to their native lands following their advanced American training. Of course, foreign-trained physicians could not be forced to return to their home countries. As Fein opined instructively, "Immigration policy is complex and involves moral issues." Fein's writings reflected the ambivalent feelings about America's increasing reliance on foreign-trained doctors, practitioners whom he characterised as "risk [y]" and "generally not as well trained" [24].

In a 1969 monograph published by Harvard University, Margulies and Bloch [25] presented a "critical review" of the subject of foreign medical graduate migration to the United States. They focused on the problem of foreign doctors who came to the United States for advanced training, then failed to return to their home countries. The authors went so far as to state that the United States had in fact done many international medical graduates (and their host countries) a favour by providing them with advanced clinical experience and a familiarity with first world technology that could be taken back with them to 'less developed' countries. Consequently, they recommended that programs should be implemented whereby foreign medical graduates were encouraged to return to their countries of origin. As Bloch and Margulies concluded presciently, "Although the poor will not become richer through better use of indigenous brain power alone, without it their prospects are very bleak indeed" [25].

### Medical migration and the 'Third World'

In 1963, it was recognized at a United Nations conference that, following World War II, poorer countries – particularly those in Asia, Africa, and Latin America – were unable to keep up with the rapid pace of scientific and technological development being witnessed in Western Europe and North America. Groups administered by the United Nations, including the World Bank, the Institute for Training and Research (UNITAR), and the Educational, Scientific and Cultural Organization (UNESCO) launched studies of the impact of skilled migration on developing countries, research investigations that were primarily economic in their scope. In 1969, there existed a growing literature investigating, or promising to address, problems of various kinds in the developing world. A relatively small portion of this literature sought to understand the 'brain drain' of professionals from underdeveloped to developed nations; an even smaller cluster of studies dealt with the impact of this 'migration of high-level manpower' on health indices. The World Health Organization's first 'multinational study' of physician and nurse migration did not appear until 1973 [21], so it is necessary to start with the earlier literature which laid the groundwork for it.

In 1970, two studies appeared which examined the international migration of skilled labourers. One, headed by F. J. Van Hoek of the Institute of Social Studies (The Hague, Netherlands), recognized that scientific and technological developments in the First World had caused a gradual shift in emphasis from 'labour-based' to 'science-based' capital formation, which meant that there was an increasing demand for skilled workers -especially engineers, scientists, and health care personnel – in the richer nations. Although it was exceedingly difficult to accurately measure the impact of the 'brain drain' on developing countries simply because of the lack of statistical data from these countries, Van Hoek suggested that detrimental effects on the development process were more or less inevitable. He vaguely called for a "better educational policy in relation to manpower needs" in both developing and developed countries [26].

The second study of 1970 was authored by the "Committee on the International Migration of Talent" (hereafter CIMT) -a cluster of economists and university professors from the United States, with representatives from the International Bank for Reconstruction and Development, the Institute of International Education, the American Association for the Advancement of Science, and Education and World Affairs (a New York-based organization that oversaw cooperative initiatives between various American Universities and countries in the developing world). Findings were presented from a two-year study (supported by the Rockefeller Foundation) that attempted to explain, region by region, the impact of the international migration of "highly-trained people". The committee recognized, somewhat defensively, that the popular use of the term "brain drain" seemed to "communicate an act of international wrongdoing". Speaking more positively of the "supply of talent" in other countries, the CIMT study suggested that the impact of health human resource migration was not so much of a problem in countries like India – one of the leading donor countries at the time – where, they opined, the annual output of that country's education system was sufficient enough to replace those physicians leaving for other parts of the world. However, in African nations such as Tanzania and Kenya, which did not have as strong an education system in place, the authors concluded that the depletion of health human resources was a much more serious matter. Like Van Hoek's work, the CIMT study lamented the lack of proper statistical information available for measuring the true impact of professional migration on the developing world [27].

One of the most acute observers of the decision of doctors to migrate to first-world countries was Oscar Gish, a European-trained health economist based in the United States. His work attempted to tell part of the story from the foreign medical graduate's perspective, while at the same time investigating the economic impact of physician emigration on poorer countries. From 1971–77, he published three important monographs. In *Doctor Migration and World Health *[28], Gish thoroughly examined each developing country in the context of its health care, providing a picture of the nuanced circumstances that contributed to a health care worker's decision to leave his or her home country. His case study of Ceylon (Sri Lanka) was especially poignant in its demonstration of the complexity of political, social, and economic factors that left, in his opinion, a medical graduate no other alternative but to leave the country in search of better opportunities [28].

Gish brought attention to the fact that the migration of health care personnel had largely been ignored by economists and other scholars in order to concentrate on other skilled groups, namely engineers and scientists. Whereas the study by the Committee on the International Migration of Talent [27] described earlier made an attempt to explain the migration of many different types of skilled labour, Gish recognized that the complicated dynamics of health care services required separate (country by country, and even region by region) scrutiny. For example, the CIMT study had suggested that India's annual educational output was large enough to supply talented manpower for both home and abroad. By erroneously clumping scientists, engineers, and medical personnel into one category, they had thereby glazed over the disparity of health care manpower between rural and urban areas. While this rural-urban divide was less of an issue for engineers and scientists (because there was not as large a demand for these professionals in rural areas), Gish observed that the rural-urban divide in health care services could not be solved by simply increasing the number of graduates a country produced annually [28].

Most importantly, Gish's work provided strong empirical basis for the eventual findings of the landmark WHO studies of the mid-1970s: that the flow of foreign-born physicians from developing countries was overwhelmingly to developed countries; and that the emigration of physicians from developed countries was overwhelmingly to other developed countries. Like Van Hoek's monograph of 1970, Gish suggested that the responsibility for this transnational migration (and the responsibility for diverting it) lay with the industrialised countries that prefer to use high-level manpower from less-developed countries because of the cost savings and the "fewer difficulties encountered if employment has to be terminated in the event of a decrease in demand or financial austerity" [28]. Here, it was understood that a developed nation's primary supply of physician manpower was from the stock that was 'home-grown'; the native medical graduate was the better trained and the preferred candidate for the job. Both Van Hoek and Gish implicitly identified an ethical problem at the individual level – namely, the viewpoint that foreign medical graduates were perceived by receiving countries to be dispensable commodities, a tapped supply to be turned on and off at will by the wealthier countries who can afford to employ them for their own needs.

Gish's contributions also included policy measures to address the impact of physician migration on developing countries. In *Health Manpower & the Medical Auxiliary *[30], Gish suggested alternative ways of increasing medical manpower in the rural areas of countries with limited economic means, mainly by training greater numbers of auxiliary personnel (as opposed to increasing the national output of physicians alone). As Gish asserted, "Manpower pyramids must be built from the bottom up" [30]. This way, a physician did not have to see every patient who presents him/herself for treatment, but a medical auxiliary could meet many of the primary needs. Many of Gish's policy and education plans from his two previous works were collected together in *Guidelines for Health Planners *[31]. These works, and others by N. R. E. Fendall [32] and Richard Smith [33], were in-depth investigations of the use of auxiliary personnel to augment doctor shortages in developing countries (shortages due in part to the emigration of medical graduates from these nations).

Late, perhaps, relative to the other studies already discussed, the World Health Organization's reports on the findings of a multinational study of physician and nurse migration [21,34-37] were landmarks (these reports were also consolidations of earlier findings over the preceding decade) in the understanding of the complex international movement of health care personnel. The final report, which combined the findings of the previous three, was published in book form in 1979, as *Physician and Nurse Migration *[37] under the lead authorship of Alfonso Mejia. Together, these reports were the first widely recognized work (the more obscure Gish and Van Hoek aside) to appreciate the complicated nature of international physician flow.

The WHO's estimates were sobering. The developing countries of the world (except the People's Republic of China, for which no data on population or physician stock was available at the time) contained two-thirds of the world's population and possessed only a quarter of the world's physicians. Moreover, nearly 90% of the world's migrant physicians were absorbed by developed countries [37]. Predictably, the poorest countries were recognized as the big losers in the international flow of physician manpower. The WHO put to rest claims made in the late 1960s by Fein, Margulies and Bloch, who asserted that additional training in "technologically advanced countries" such as the United States or Canada was beneficial to less developed countries. According to the WHO, even if most foreign-born physicians did return home, physicians were not likely to benefit their home countries with their new-found skills and experience simply because of the disparity in health infrastructures and the technology gap between developed and developing countries [36].

According to Stephen Bach [39], the 1979 World Health Organization monograph by Mejia et al. was the definitive and most detailed analysis on the migration patterns of physicians and nurses in the 1960s and 1970s. Mejia and his team were the first to articulate a "relationship between GDP, the production of physicians, and the likelihood that they would emigrate" and, statistical correlations aside, the WHO study was the first to be concerned with the international implications of skilled-labour migration. Of course, as we have seen, scholars like Gish and Van Hoek were instrumental in laying the groundwork for the WHO studies that make up the 1979 monograph. However, Mejia et al.'s work has been the most influential on subsequent monographs, including reports on the trends and policy implications for international nurse mobility [38] and the social issues surrounding the international migration of health workers [38,39].

### Western bioethics meets global social justice

There appear to be several reasons why the migration of physicians from developing to developed countries did not coalesce in the 1960s and early 1970s into a major ethical debate. The first concerns the unpredictable movement of the physicians themselves. While plenty of statistical information was available regarding the inflow of migrating physicians to wealthy countries, the outflow (emigration) records of developing countries were fragmented, if they existed at all. Social scientists had to, in effect, piece together the larger puzzle by working backwards from data in recipient nations. In addition, this poorly understood drain of resources was supposed by most scholars to be only temporary. It was frequently assumed that many foreign-born or trained physicians who had migrated would eventually return home following a period of additional or "advanced training" in the developed country [30]. The reality that most of these physicians settled abroad permanently or migrated to yet other developed countries was not widely recognized. The degree and nature of permanent international migration of physicians from poorer to richer countries had to be determined before it could be assessed or judged to be explicitly unethical.

Complicating matters, as Gish first demonstrated, was the phenomenon of certain developed countries – like Britain and Canada – being in the then top nations as both donor *and *recipient countries, owing to their status as countries used as "stepping stones" to elsewhere. In these cases, nations simultaneously received physicians from abroad while they themselves were losing health human resources to medical migration elsewhere. Further, there were a small number of countries who began *supporting *the migration of physicians either for geo-political, historical or financial reasons. Castro embarked on a self-conscious policy of training physicians for export, in order to support the ideal of socialized medicine and socialist politics. Ireland, a country that had a long history of out-migration reconciled itself to the fact that many physicians would leave for elsewhere by generating a capacity to graduate more doctors than the country could absorb. Finally, in a manner analogous to its support of nurse and caregiver migration [13], the Philippines appears to have encouraged the out-migration of physicians in order to facilitate the flow of millions of dollars of remittances.

Cultural attitudes and racial prejudices also played an important role. Most scholars of the 1960s and 1970s viewed foreign-born and -trained physicians -especially those from underdeveloped nations – as inferior to the medical graduates from developed countries. In other words, most early sources that investigate doctor migration demonstrate a prejudice towards the quality of care provided by foreign medical graduates, even though many countries were relying heavily on these foreign workers to fill the gaps in their health service systems. It took a good decade for international medical graduates to prove themselves worthy of being considered and discussed as equals with physicians trained in industrialized countries. Only then could the ethical problems begin to be articulated with respect to the drain of medical personnel from developing countries to developed ones. Foreign physicians had to be appreciated as being valuable resources before ethical, transnational concerns could be conceived of, and applied to, their situation.

Finally, there seems to have been an implicit understanding that it was ultimately the physician's choice to leave his or her home country -which removed culpability from developed countries for 'stealing' medical personnel. As health economist Alfonso Mejia put it rather elegantly, "Learned men (and women) have always travelled abroad seeking a more congenial intellectual milieu to realise their full potential" [36]. The physician's decision to migrate was understood to be a very personal calculation based upon a unique set of factors for each individual, the prevention of which would itself be both impossible and unethical. How could the post-war Western World embrace refugees and economic migrants but deny the right of educated individuals to better their personal situations? Oscar Gish described both sides of the moral dilemma for the physician migrating from developing countries in his discussion of the term 'brain drain':

The term itself conjures up images of highly sophisticated men (and women) who choose to work in countries other than those in which they were born. Because of such images feelings of great loss may be held by countries being 'drained of brains'. On the other hand...spirited defences of the free movement of great men (and women) are made in the name of freedom as well as in the interest of maximizing the output of world science and/or economic output.

Thus even those who were coming to understand the magnitude of the problem recoiled from suggesting interventionist measures to stop it. There appeared to be a conceptual chasm: how could thousands of defensible individuals moral decisions constitute one large collective ethical problem?

The conceptual leap – from an individualistic bioethics attitude which framed ethical issues within the doctor-patient-relationship to one that began to conceptualize collective rights and identify problems of global social justice – was a long time in formation. Bioethics was, for the longest time, rooted in moral dilemmas arising from the increasing use of medical technologies particularly within North American and Western settings [50]. Bioethics was thus preoccupied with micro-ethical issues and has only recently begun to focus on what are increasingly called global health ethics. This intellectual shift is reflected in the growing use of the term 'global health' in medical literature, one which took off in the 1990s as a term to replace international health. As Brown et al. [51] explain, global health in contradistinction to "international health...recognizes the growing importance of actors beyond governmental or intergovernmental organizations and agencies". The transnational migration of health workers clearly falls within this 'global' realm and outside the traditional discourses of Western bioethics.

### Out of Africa

During the 1980s and early 1990s, the interest in the international migration of foreign-trained doctors subsided in concert with the dramatic decline in the licensing of IMGs in most Western Countries. By the late 1990s, however, the issue of national doctor and nursing shortages had emerged as a major topic of concern and public interest. By this time, rural regions of industrialized countries were finding themselves denuded of primary care and looked abroad to foreign-trained doctors as a solution [13,14]. For the last decade then, western countries have ramped up their licensing of foreign-trained doctors. But, unlike a generation ago an ethical and public policy debate has emerged around this phenomenon. In this current era of globalization, politicians and policy makers could no longer claim ignorance about the impact of medical migration on donor countries.

Within the new ethical debate, South Africa has played a totemic role [43-49]. Devastated by the AIDS pandemic and struggling with rebuilding a post-apartheid civil society, the dramatic exodus of (mainly) white doctors, aided and abetted by western countries, has touched raw nerves. The exact number of doctors who are practising abroad is unknown, but the South African Medical Association estimated in 2002 that over 3,500 (approximately 50%) of its domestically-trained doctors were living abroad [48]. Ironically, South Africa itself backfills, by recruiting African doctors from poorer states, such as Uganda and Tanzania. The *South African Medical Journal *describes a "medical carousel", in which doctors seem to be continually moving to countries with a perceived higher standard of living [1].

The ethical debate currently revolves around several related issues. Critics point to the purposeful underproduction of health human resources in the West to be supplemented, as a matter of policy, by foreign medical graduates. This results, they argue, in the depletion of health human resources in countries that are not only poorer but often plagued by serious public health challenges. On the opposite side, commentators suggest that it would be unethical to restrict the free movement of skilled labour in an era of globalization. Physicans, they argue, have as much a right to safe working conditions, or decent pay, as anyone else does [1-9]. Some have even argued that the poor public health conditions in Africa are "a result of factors unrelated to international movement of health professionals" [44,45]. Others lament that, even if a restriction on the emigration of health professionals may be desirable, it would be largely impossible to enforce. Nevertheless, the embarrassing optics of rich countries exploiting the health human resources of African countries devastated by the AIDS epidemic – one that has unfortunate trappings of neo-colonialism – has led to some intermittent policy initiatives. Britain, for example, recently pledged to tighten the loopholes in its three-year old commitment to stop recruiting from the 'developing world' [52,53].

The solution is not at all straightforward as the causes of physician (and nurse) migration are not uniform. A combination of better pay, better (and safer) working conditions, fewer patients on a caseload, national public health policies, political instability, personal safety, epidemics, and future prospects, are only some of the reasons given. Indeed, rather than trying to reverse the brain drain, which critics suggest is impossible, inconsequential, impractical or (itself) ethically problematic, some organizations have suggested two things: an increase of domestic supply of physicians and compensatory schemes from recipient to donor countries. An increase of the domestic supply of physicians (in industrialized countries) would, some argue, relieve the pressure on the recruitment of foreign-trained doctors. Compensatory schemes are more radical. In these scenarios recipient countries would compensate the countries where the physician trained either in straight monetary terms or through medical exchange programs [1-8]. These transfers would assist in enhanced physician remuneration that would reduce one major factor in the decision to emigrate. Such a system, however, would necessitate a strong international organization with the ability to enforce rules and determine levels of compensation.

At the 2005 World Health Assembly, the WHO resolved that World Health Day in 2006 should focus on the crisis of international migration of health personnel. Additionally, the assembly determined that their General Programme of Work, 2006–2015 should focus on the complexity of issues involved in international health human resource migration. Yet, despite recognition at the highest public policy levels, the question of physician migration and recruitment has failed to gain much traction from the lay public. Perhaps there is little popular appeal in industrialized countries to solutions that may, in many unpredictable ways, make the complex problem of doctor shortages worse. And so the brain drain continues and threatens to worsen over the next decade. As this article has demonstrated, the current wave of international physician migration, accelerated in part by health policy and immigration decisions made in industrialized countries, has a longer history to it than many current scholarly articles acknowledge. An historical perspective assists us in understanding the broader social and economic forces at work, as well as the changing ethical framework within which we understand this complex issue facing the world today.

## Authors' contributions

All authors contributed equally to the manuscript.

## Competing interests

The authors declare that they have no competing interests.
